# Clinical usefulness of 16S ribosomal RNA real-time PCR for the diagnosis of scrub typhus

**DOI:** 10.1038/s41598-021-93541-w

**Published:** 2021-07-12

**Authors:** Na Ra Yun, Choon-Mee Kim, Da Young Kim, Jun-Won Seo, Dong-Min Kim

**Affiliations:** 1 Department of Internal Medicine, College of Medicine, Chosun University, Gwangju, Republic of Korea; 2 Premedical Science, College of Medicine, Chosun University, Gwangju, Republic of Korea; 3 Department of Internal Medicine, School of Medicine, Chosun University, 588 Seosuk-dong, Dong-gu, Gwangju, 501-717 Republic of Korea

**Keywords:** Microbiology, Diseases

## Abstract

Scrub typhus is a major acute febrile disease in the Asia–Pacific region. The purpose of the present study is to investigate the clinical usefulness of real-time PCR (Q-PCR) of 16S rRNA for the diagnosis of scrub typhus. We examined blood specimens from 148 adult patients who were confirmed to have scrub typhus from September 2008 to December 2009. Among the 148 scrub typhus patients, 36 patients were treated with antibiotics before admission. To evaluate the clinical usefulness of 16S rRNA Q-PCR, we compared its diagnostic accuracy to the accuracy of the following methods: nested PCR (N-PCR) targeting the gene encoding the 56-kDa protein, Q-PCR targeting the gene encoding the 47-kDa protein, and conventional PCR (C-PCR), targeting the 16S rRNA gene. According to 16S rRNA Q-PCR and 47-kDa Q-PCR, the mild group had copy numbers of 234.4 ± 261.9 and 130.5 ± 128.3, whereas the severe group had copy numbers of 584.4 ± 911.4 and 244.7 ± 210.9, respectively. In both tests, the mean copy numbers were significantly greater in the severe group (P = 0.037 and P = 0.035). 16S rRNA Q-PCR detected *Orientia tsutsugamushi* infections with a sensitivity of 91.9% (95% CI 86.3–95.7), and 56-kDa N-PCR, 47-kDa Q-PCR, and 16S rRNA C-PCR exhibited lower sensitivities of 81.1% (95% CI 73.8–87.0), 74.3% (95% CI 66.5–81.1), and 87.8% (95% CI 81.5–92.6), respectively, for all 148 patients. In addition, 16S rRNA Q-PCR exhibited a sensitivity of 99.1% (95% CI 95.1–100.0) in the 112 patients who were not treated with antibiotics before admission. 16S rRNA Q-PCR is clinically useful for the rapid diagnosis of scrub typhus and is more accurate than the 56-kDa N-PCR, 47-kDa Q-PCR, and 16S C-PCR methods.

## Introduction

Scrub typhus is an acute febrile disease, caused by bites from trombiculid mites infected by *Orientia tsutsugamushi*^1,2^. In most cases, scrub typhus presents with manageable clinical symptoms and positively responds to treatment with antibiotics; however, in cases of late diagnosis, serious complications can arise and possibly lead to multi-organ failure and death^1^. Thus, for the successful treatment of scrub typhus, a timely diagnosis made in the early stages is very important. Various serological tests, including indirect immunofluorescence assay (IFA), immunoperoxidase test, enzyme-linked immunosorbent assay, and passive hemagglutination test, have been used to diagnose scrub typhus. However, these tests exhibit low sensitivity in the early stages of the disease due to the low production of antibodies, and retests are therefore required in periods of convalescence for accurate diagnosis; consequently, these methods are not clinically useful^3,4^. These limitations demonstrate the need for diagnostic tests that can provide an accurate diagnosis in the early stages of scrub typhus. Therefore, polymerase chain reaction (PCR) conducted with genes specific to *O. tsutsugamushi* found in the blood of scrub typhus patients has been investigated as a diagnostic test for scrub typhus. In particular, conventional PCR (C-PCR), nested PCR (N-PCR), and real-time PCR (Q-PCR) have become useful tests for diagnosis^5,6^. Notably, Q-PCR yields faster results than C-PCR or N-PCR, exhibits high sensitivity and specificity, and produces quantitative results^7^. In PCR tests conducted for the diagnosis of scrub typhus, the genes encoding the 56-kDa and 47-kDa proteins have been used as specific biomarkers. Furthermore, 16S ribosomal RNA (16S rRNA), which has a long length of 1550 bp, has often been used for bacterial identification. The clinical usefulness of C-PCR conducted with 16S rRNA for the diagnosis of scrub typhus has also been investigated previously^5,6^.

The purpose of the present study was to investigate the clinical usefulness of Q-PCR of 16S rRNA (16S rRNA Q-PCR) for the diagnosis of scrub typhus compared with that of other tests that are known to be useful for the diagnosis of scrub typhus: N-PCR for the gene that encodes the 56-kDa protein (56-kDa N-PCR), Q-PCR for the gene that encodes the 47-kDa protein (47-kDa Q-PCR), and C-PCR for 16S rRNA (16S rRNA C-PCR). This study compared the sensitivity of these four tests in patients diagnosed with scrub typhus and investigated how the use of antibiotics and the severity of the condition can influence the tests. Furthermore, considering that Q-PCR can yield quantitative results, this study aimed to confirm whether the copy number is associated with the severity of scrub typhus or with complications. Ultimately, we sought to determine the clinical usefulness of 16S rRNA Q-PCR.

## Materials and methods

### Patient selection

Total 148 patients were enrolled in the study. Tests were conducted using the same blood samples collected from 148 patients over 18 years of age who were diagnosed with scrub typhus at the Chosun University Hospital between September 2008 and December 2009. Patients that exhibited more than fourfold increases in IgM or IgG titer on IFA were defined as confirmed cases.

### Definitions

Those who were not administered any antibiotics before the visit or those administered antibiotics, such as beta-lactam or aminoglycoside, with no confirmed effect on scrub typhus were defined as patients without prior antibiotics treatment. Those administered doxycycline, rifampin, azithromycin, ciprofloxacin, or levofloxacin were defined as patients with prior antibiotics treatment.

The severity was scored out of 6 (total score: 0–6) by adding a score of 1 for the presence of each of the following symptoms: admission into the intensive care unit, hepatic damage (alanine aminotransferase > 100 U/L), pulmonary damage (arterial oxygen saturation < 70 mmHg), renal failure (creatinine ≥ 1.8 mg/dL), hematological abnormalities (platelet count < 120,000/µL), and neurological symptoms (confusion)^8^. Severity was sub-grouped into 4 levels: level 0 for a score of 0, level 1 for a score of 1, level 2 for a score of 2, and level 3 for a score of 3 or higher. Severity levels 0 and 1 were grouped together into the mild group, and severity levels 2 and 3 were grouped together into the severe group.

The duration of symptoms refers to the number of days between the onset of scrub typhus symptoms, which included fever, chills, and myalgia, and the visit to our hospital.

### Polymerase chain reaction (PCR)

#### 16S rRNA gene PCR

**Figure 1:**
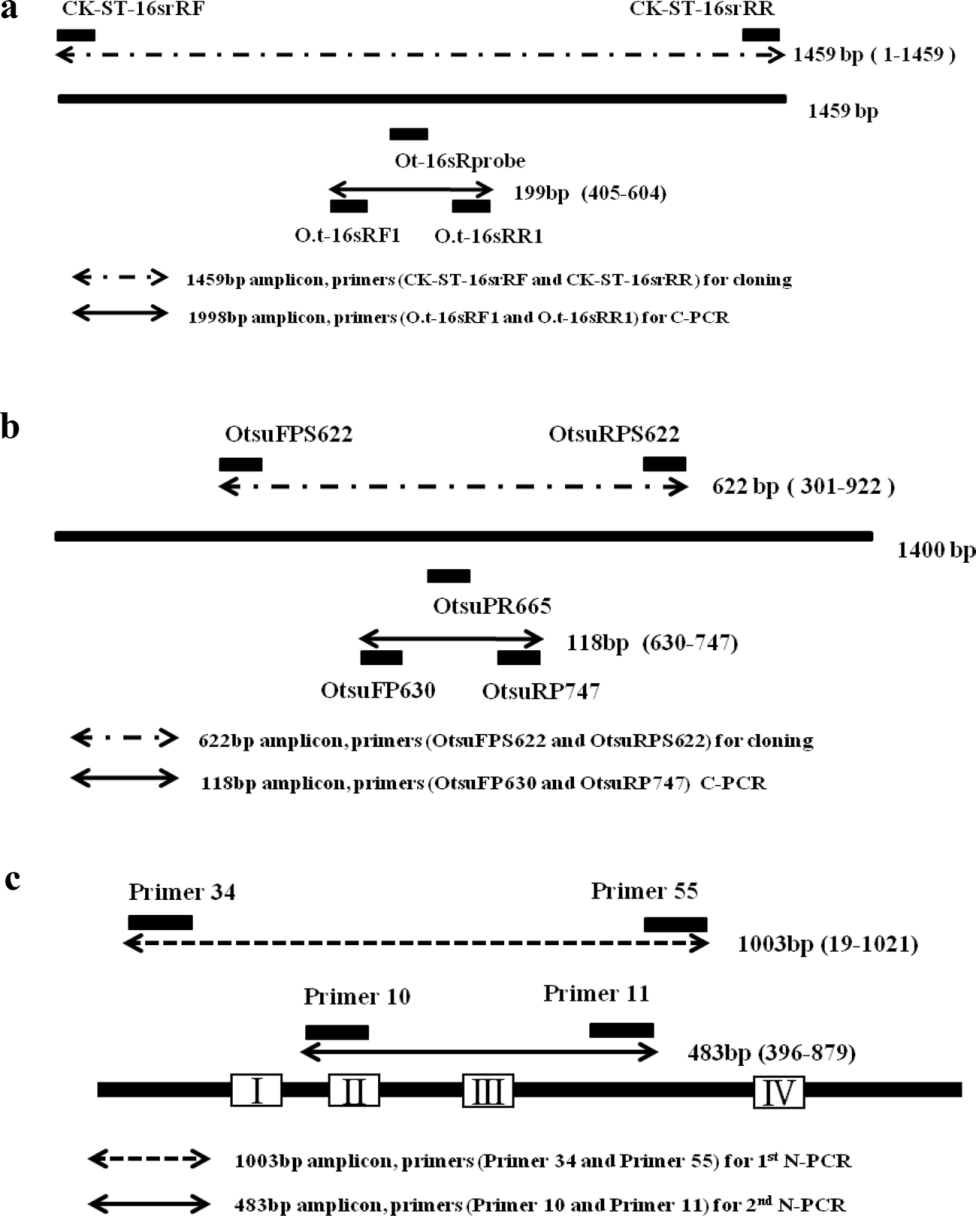
Diagram indicating the primer positions in *Orientia tsutsugamushi*. (**a**) 16S rRNA gene. (**b**) 47-kDa protein gene. (**c**) 56-kDa protein gene.

The primers and the probe used were those for the 16S rRNA sequence of the *O. tsutsugamushi* Gilliam strain (accession no. L36222) as described previously^9^. For the probe, 6-carboxyfluorescein (FAM) was attached to the 5′ end, and black hole quencher 1 (BHQ-1) was attached to the 3′ end (Fig. 1).

The standard curve was generated with tenfold serial dilutions of 10^8^ copies/μL of plasmid DNA prepared by cloning partial DNA fragments of the *O. tsutsugamushi* 16S rRNA gene. A total volume of 20 μL of reaction solution was prepared for Q-PCR by mixing 5 μL of DNA, 1 μL (5 pmol/μL) of each primer, 1 μL (2 pmol/μL) of the probe, 4 μL of 5× master mix (reaction buffer, Fast Start Taq DNA polymerase, MgCl_2_, and deoxynucleoside triphosphates (with dUTP instead of dTTP), and sterilized triple-distilled water. For the PCR, an initialization step was conducted at 90 °C for 10 min followed by 45 cycles of 2-step reactions conducted for 10 s at 95 °C and for 30 s at 60 °C; final cooling was subsequently conducted at 40 °C for 30 s. The Q-PCR results were analyzed with the Light Cycler software, version 4.0 (Roche, Basel, Switzerland). Cases with a crossing-point value less than 38 were considered positive, as described previously^9^.

#### 47-kDa real-time PCR

Probe OtsuPR665 and OtsuFP630 and OtsuRP747 primers previously described by Jiang et al. were used^10^. For the probe, 6-carboxyfluorescein (FAM) was attached to the 5′ end and black hole quencher 1 (BHQ-1) was attached to the 3′ end (Fig. 1)^5,10^.

The standard curve was generated with tenfold serial dilutions of 10^8^ copies/μL of plasmid DNA prepared by cloning partial DNA fragments of the *O. tsutsugamushi* gene that encodes the 47-kDa protein. Q-PCR was conducted after preparing 20 μL of reaction solution by mixing 5 μL of DNA, 1 μL (5 pmol/μl) of each primer, 1 μL (2 pmol/μL) of the probe, 4 μL of 5× master mix (reaction buffer, Fast Start Taq DNA polymerase, MgCl_2_, and deoxynucleoside triphosphates [with dUTP instead of dTTP]), and sterilized triple-distilled water. For PCR, an initialization step was conducted at 95 °C for 10 min, followed by 45 cycles of a 2-steps reaction conducted of 95 °C for 10 s and at 60 °C for 30 s; the final cooling was subsequently conducted at 40 °C for 30 s. The results of the Q-PCR were analyzed using the LightCycler software package, version 4.0 (Roche, Basel, Switzerland). Cases with a crossing-point value less than 38 were considered positive.

#### 56-kDa nested PCR

We used the primers described by Furuya et al. for the *O. tsutsugamushi* Gilliam strain gene encoding the 56-kDa protein (Fig. 1)^11^. Nested PCR was performed by a method described previously^12^.

### Data analysis

The sensitivity was analyzed by calculating the positive rate of diagnosis for each PCR test method. Student’s t-test was performed to compare the viral copy number and the complications of severity groups. Further, to compare the viral copy numbers of each (four) severity groups, analysis of variance (ANOVA) were conducted. If the ANOVA results showed significant differences, the Scheffe post hoc analysis was used for post‐analysis. Pearson's correlation coefficients were calculated to establish the correlation between them. McNemar tests were performed to determine the statistical differences in sensitivity across two variables. The null hypothesis tested here to evaluate whether the marginal probability of the two targeted PCR for each outcome (sensitivity) are the same or whether there is any significant difference between them. For all statistical analyses, statistical significance was defined as p-values less than 0.05. The SAS software package, version 8.2 (SAS Institute Inc., Cary, NC), was used for statistical analysis.

### Ethics statement

This study was approved by the Institutional Review Board (IRB Number—201310004) of Chosun University Hospital. The patients provided written informed consent to participate in the study. All methods were performed in accordance with the relevant guidelines and regulations.

## Results

### Sensitivity of each PCR test

#### For all patients diagnosed with scrub typhus

**Figure 2:**
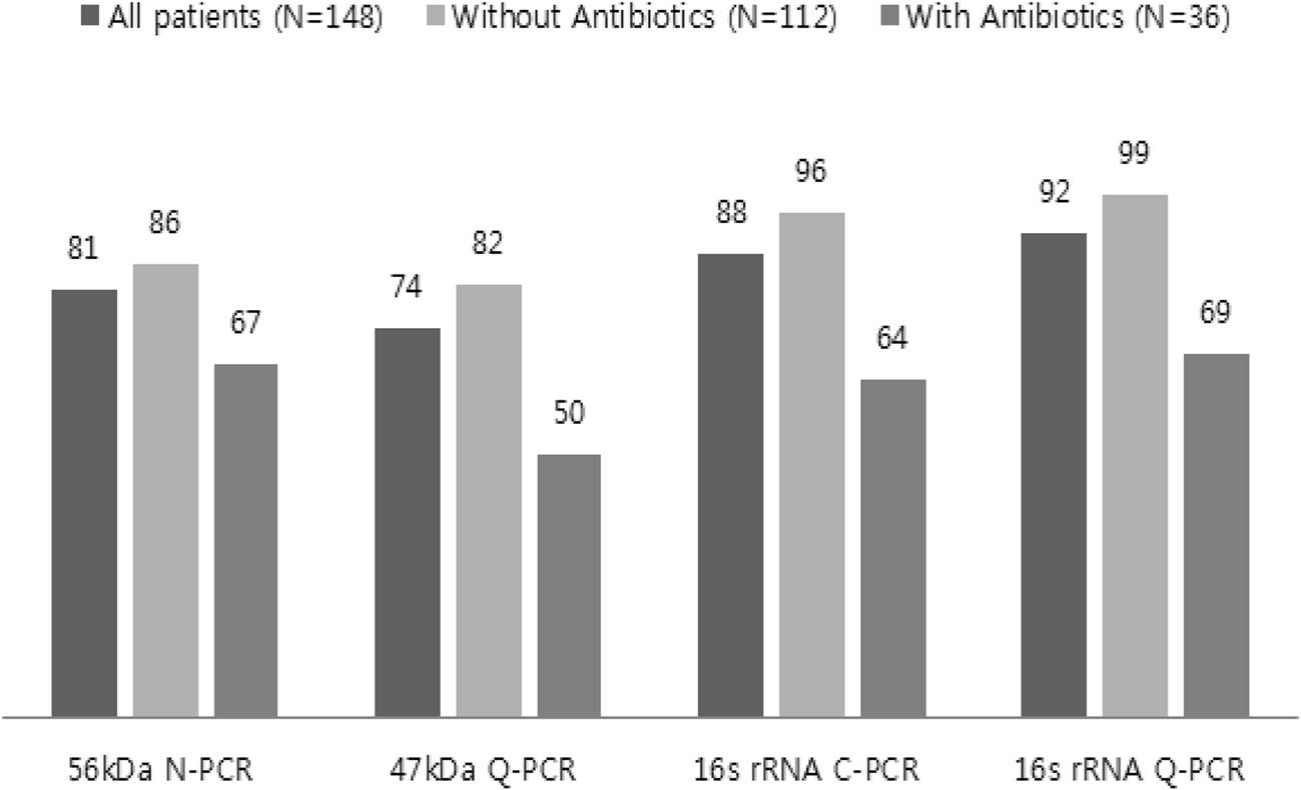
Sensitivities (%) of 56-kDa N-PCR, 47-kDa Q-PCR, 16S rRNA C-PCR, and 16S rRNA Q-PCR for three groups: all patients, patients without prior antibiotics treatment, and patients with prior antibiotics treatment.

56-kDa N-PCR, 47-kDa Q-PCR, 16S rRNA C-PCR, and 16S rRNA Q-PCR were conducted using blood samples collected from 148 patients with a confirmed diagnosis of scrub typhus; the positive rates were 81.1% (95% CI 73.8–87.0), 74.3% (95% CI 66.5–81.1), 87.8% (95% CI 81.5–92.6), and 91.9% (95% CI 86.3–95.7), respectively. Of the 4 tests, 16S rRNA Q-PCR exhibited the highest sensitivity (Fig. 2). McNemar tests were conducted for all patients diagnosed with scrub typhus in order to compare the sensitivities of the four different kind of PCR. We compare the sensitivities of the 4 tests; 47-kDa Q-PCR and 56-kDa N-PCR did not exhibit any significant difference, whereas 16S rRNA C-PCR and 16S rRNA Q-PCR exhibited significantly higher sensitivities than 56-kDa N-PCR (P = 0.03, P < 0.001, respectively). 16S rRNA C-PCR and 16S rRNA Q-PCR had significantly higher sensitivities than 47-kDa Q-PCR (P < 0.001, P < 0.001, respectively). Moreover, the sensitivity of 16S rRNA Q-PCR was significantly greater than that of 16S rRNA C-PCR (P = 0.01).

#### According to the previous use of antibiotics

**Figure 3:**
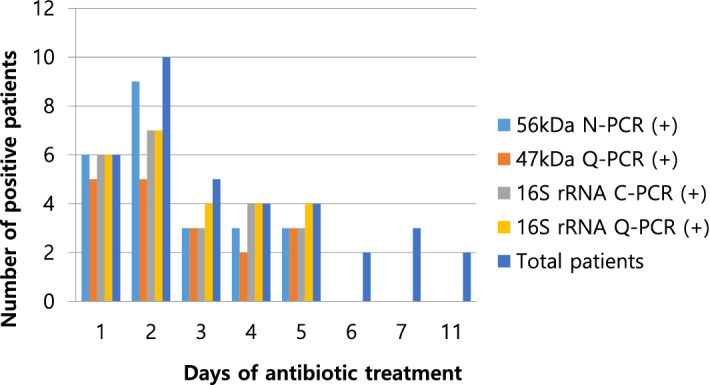
Number of positive patients for the four PCR methods according to the days of antibiotics treatment in 36 patients with prior antibiotics treatment.

The sensitivity of each test was investigated for patients who received prior antibiotics treatment and those who had not. For the 112 patients without prior antibiotics treatment, the positive rates observed were 85.7% (95% CI 77.8–91.6), 82.1% (95% CI 73.8–88.7), 95.5% (95% CI 89.9–98.5), and 99.1% (95% CI 95.1–100.0) for 56-kDa N-PCR, 47-kDa Q-PCR, 16S rRNA C-PCR, and 16S rRNA Q-PCR, respectively; 16S rRNA Q-PCR exhibited the highest sensitivity (Fig. 2). For those with prior antibiotics treatment, the positive rates obtained using 56-kDa N-PCR, 47-kDa Q-PCR, 16S rRNA C-PCR, and 16S rRNA Q-PCR were 66.7% (95% CI 49.0–81.4), 50.0% (95% CI 32.9–67.1), 63.9% (95% CI 46.2–79.2), and 69.4% (95% CI 51.9–83.7), respectively, with 16S rRNA Q-PCR exhibiting the highest sensitivity (Fig. 2). The length of pre-visit antibiotics treatment varied from 1 to 11 days in the 36 patients, and the differences in the positive rate that were observed were correlated with the length of treatment (Fig. 3).

### Comparison of the copy number detected using 47-kDa Q-PCR and 16S rRNA Q-PCR according to the disease severity and presence of complications

Our findings confirmed that the copy number of each Q-PCR test increased with the severity of scrub typhus. To exclude the effects of antibiotics on Q-PCR, an analysis was conducted only on the 112 patients without prior antibiotics treatment. Those who had positive results on 16S rRNA Q-PCR and 47-kDa Q-PCR were divided into 4 severity groups, and the mean copy number of each severity group was compared using ANOVA. Using 16S rRNA Q-PCR, the mean copy numbers were 255.0 ± 324.6, 218.4 ± 203.3, 446.8 ± 711.7, and 900.8 ± 1247.1, whereas they were 128.9 ± 151.7, 131.8 ± 108.0, 184.5 ± 164.3, and 296.9 ± 271.0 when 47-kDa Q-PCR was used; in both tests, the mean copy numbers increased significantly as the severity of scrub typhus increased (P = 0.003 and P = 0.019, respectively) (Table 1). Moreover, the 112 patients without prior antibiotics treatment were divided into the mild and severe groups and compared. The mean copy numbers from the 16S rRNA Q-PCR and 47-kDa Q-PCR results for the mild group were 234.4 ± 261.9 and 130.5 ± 128.3, respectively, whereas the severe group had copy numbers of 584.4 ± 911.4 and 244.7 ± 210.9, respectively; in both tests, the mean copy numbers were significantly greater in the severe group (P = 0.037 and P = 0.035, respectively) (Table 2). The copy numbers obtained from 47-kDa Q-PCR and 16S rRNA Q-PCR for the following complications were compared through t-tests: hemorrhage, pneumonia, hepatic malfunction, and renal malfunction. The copy numbers obtained using both16S rRNA Q-PCR and 47-kDa Q-PCR were significantly greater when pneumonia was present (P = 0.05 and P = 0.013, respectively) (Table 3). Furthermore, to investigate the correlation between the copy numbers obtained using 47-kDa Q-PCR and 16S rRNA Q-PCR and the various test results; Pearson's correlation coefficients were calculated. 47-kDa Q-PCR exhibited significant correlations with decreases in white blood cell count (WBC), C-reactive protein (CRP), and albumin (P = 0.001, P = 0.005, and P < 0.001, respectively), whereas 16S rRNA Q-PCR exhibited significant correlations with decreases in WBC, C-reactive protein (CRP), creatinine, and albumin (P = 0.003, P = 0.014, P = 0.009, and P < 0.001, respectively) (Table 4).

**Table 1:** Mean copy number detected by 16S rRNA Q-PCR and 47-kDa Q-PCR according to the severity grade divided into four levels.

Severity	16S rRNA Q-PCR		Post hoc *p* value	47-kDa Q-PCR		Post hoc *P* value
	Number of patients	Copy number Mean ± SD		Number of patients	Copy number Mean ± SD	
0^a^	34	255.0 ± 324.6	0.013^a,d^ 0.005^b,d^	29	128.9 ± 151.7	0.041^a,d^ 0.039^b,d^
1^b^	44	218.4 ± 203.3		36	131.8 ± 108.0	
2^c^	23	446.8 ± 711.7		18	184.5 ± 164.3	
3^d^	10	900.8 ± 1247.1		10	296.9 ± 271.0	
ANOVA *P* value	0.003			0.019		

*SD* standard deviation.

**Table 2:** Mean copy number detected using 16S rRNA Q-PCR and 47-kDa Q-PCR according to the severity grade divided into 2 levels.

Severity	16S rRNA Q-PCR		47-kDa Q-PCR	
	Number of patients	Copy number Mean ± SD	Number of patients	Copy number Mean ± SD
Mild	78	234.4 ± 261.9	65	130.5 ± 128.3
Severe	33	584.4 ± 911.4	28	224.7 ± 210.9
*P* value	0.037		0.035	

‘Number of Patients’ indicates the number of positive patients detected by each PCR method for patients without prior antibiotics treatment.

*SD* standard deviation.

**Table 3:** Mean copy number detected by 16S rRNA Q-PCR and 47-kDa Q-PCR according to complications.

	16S rRNA Q-PCR		47-kDa Q-PCR	
	Number of patients	Copy number Mean ± SD	Number of patients	Copy number Mean ± SD
**Hemorrhage**				
No	106	348.3 ± 572.6	90	161.1 ± 164.6
Yes	5	129.9 ± 101.6	3	91 ± 23.6
*P* value	0.39		0.47	
**Pneumonia**				
No	99	269.5 ± 445.6	81	130.8 ± 123.7
Yes	12	907.1 ± 994.8	12	348 ± 254
*P* value	0.05		0.013	
**Renal impairment**				
No	101	307.9 ± 214.5	84	154 ± 159.9
Yes	10	647.1 ± 1119.9	9	204.5 ± 188.7
*P* value	0.366		0.378	
**Hepatic injury**				
No	8	567.7 ± 1642.1	7	182.4 ± 235.3
Yes	103	320.6 ± 471.6	86	156.9 ± 156.9
*P* value	0.605		0.692	

‘Number of patients’ indicates the number of positive patients detected by each PCR method for patients without prior antibiotics treatment.

*SD* standard deviation.

**Table 4:** Correlation between laboratory findings and copy number determined using 47-kDa Q-PCR and 16S rRNA Q-PCR.

	47-kDa real time PCR		16s rRNA real-time PCR	
	Correlation coefficient	*P* value	Correlation coefficient	*P* value
WBC	0.312	0.001	0.256	0.003
Platelet	− 0.166	0.082	− 0.104	0.224
CRP	0.265	0.005	0.212	0.014
Creatinine	0.104	0.279	0.224	0.009
Albumin	− 0.379	< 0.001	− 0.36	< 0.001
Bilirubin	0.149	0.119	0.013	0.881
APACHE II score	0.20	0.06	0.14	0.14

*WBC* white blood cell, *CRP* C-reactive protein.

### Comparison of PCR positive rates according to the duration of symptoms

**Figure 4:**
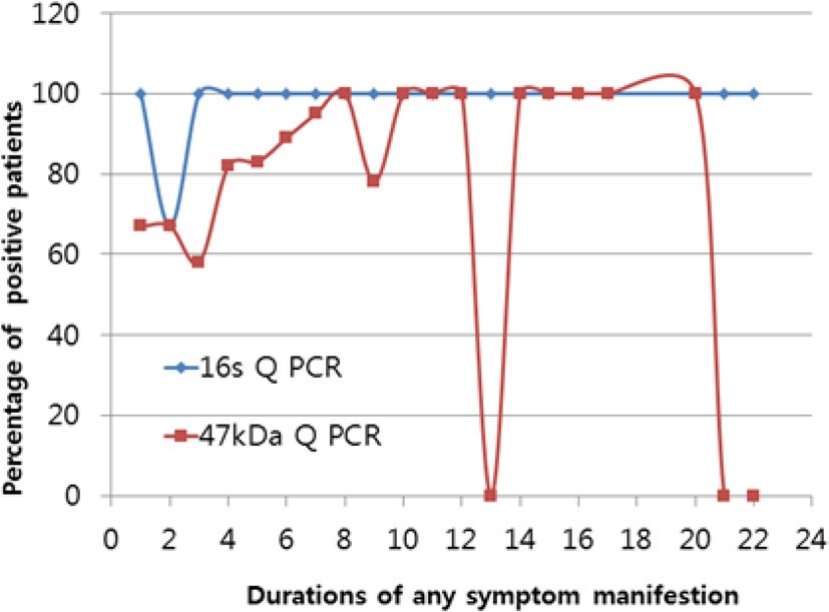
Positivity percentages of 47-kDa real-time PCR and 16s rRNA real-time PCR according to the durations of symptom manifestation.

The positive rates of 16S rRNA Q-PCR and 47-kDa Q-PCR were investigated in the 112 patients without prior antibiotics treatment according to the time between the onset of symptoms and the time of hospital visit. The 47-kDa Q-PCR results exhibited a low positive rate on day 3 after the onset of symptoms; the rate increased starting on day 4 and reached 100% between days 8 and 12. The rate decreased thereafter. 16S rRNA Q-PCR yielded negative results for only 1 patient tested on day 2 after the onset of symptoms and yielded positive results for all other patients, including the patient with the longest duration of symptoms, 21 days (Fig. 4).

## Discussion

Studies have investigated the clinical usefulness of PCR for the timely diagnosis of scrub typhus. One study conducted PCR for 16s rRNA in scrub typhus patients and reported 44.8% sensitivity and 99.7% specificity; another study reported 50.0% sensitivity and 99.0% specificity^6,7^. In the present study, sensitivities of 81.1% (95% CI 73.8–87.0), 74.3% (95% CI 66.5–81.1), 87.8% (95% CI 81.5–92.6), and 91.9% (95% CI 86.3–95.7) were observed for 56-kDa N-PCR, 47-kDa Q-PCR, 16S rRNA C-PCR, and 16S rRNA Q-PCR, respectively, conducted on a total of 148 patients; the sensitivity was higher in our tests compared with those of previous studies. N-PCR exhibited a much higher sensitivity, often more than 100-fold higher, than C-PCR.Q-PCR yields results within 2 h. All PCR tests are influenced by antibiotics treatment^10,13^. In a study by Kim et al., the sensitivity of the 56-kDa N-PCR conducted for the diagnosis of scrub typhus was 90.5% before the administration of antibiotics; however, the sensitivity decreased to 60.5% and 10% after 3 and 4 days of antibiotics treatment, respectively, thus indicating that PCR should be conducted within 3 days of antibiotics administration for the diagnosis of scrub typhus^13^. In addition, in the present study, the patients were divided into those with and those without prior antibiotics treatment to compare the sensitivity of each PCR method according to the use of antibiotics. In 112 patients without prior antibiotics treatment, sensitivities of 85.7% (95% CI 77.8–91.6), 82.1% (95% CI 73.8–88.7), 95.5% (95% CI 89.9–98.5), and 99.1% (95% CI 95.1–100.0) were obtained using 56-kDa N-PCR, 47-kDa Q-PCR, 16S rRNA C-PCR, and 16S rRNA Q-PCR, respectively; in particular, even though 16S rRNA Q-PCR exhibited a high sensitivity, yielding positive results for all except 1 patient, the sensitivity decreased significantly for patients with prior antibiotics treatment. When PCR was conducted within 3 days of antibiotics treatment, sensitivities of 90%, 61.9%, 76.2%, and 81.0% were observed using 56-kDa N-PCR, 47-kDa Q-PCR, 16S rRNA C-PCR, and 16S rRNA Q-PCR, respectively; when the tests were conducted after 4 days of antibiotics treatment, the sensitivities decreased to 40%, 33.3%, 46.7%, and 53.3%, respectively. Previously, the specificity study was also performed for the targeted 56-N-PCR, 16-C-PCR, and 47-Q-PCR that yield 100% diagnostic and analytical specificity^5,9^. In particular, all four PCR tests yielded negative results after 6 days of antibiotics treatment, indicating that PCR should be conducted after no more than 6 days of antibiotics treatment for diagnosis. 16S rRNA Q-PCR exhibited the highest sensitivity for both patients with and without prior antibiotics treatment. Although it would be preferable to conduct PCR for the diagnosis of scrub typhus before the administration of antibiotics, if it is necessary to perform the test after antibiotics administration, 16S rRNA Q-PCR, rather than other PCR tests, is more useful for diagnosis. 16S rRNA Q-PCR yielded a negative result for one patient without prior antibiotics treatment; the crossing-point (Cp) value was 40, and the copy number was 20. Mild symptoms, including myalgia, appeared in this particular patient 2 days before presentation, and fever was observed on the day of presentation. The symptoms were very mild in general and improved after 3 days of hospitalization, after which the patient was discharged. At the time of PCR, the symptoms had been present for only a few days and were very mild, which may have led to the false-negative results obtained via PCR.

Because the results of Q-PCR can be quantitatively analyzed, multiple studies have investigated this aspect; a study analyzing the 16S rRNA gene in scrub typhus patients reported that the DNA load was significantly higher in those who died than in those who survived^14^. In addition, in the present study, the relationship between the DNA load and the severity of scrub typhus was investigated using 16S rRNA Q-PCR. Although this study did not investigate the correlation with death, the study still found that the DNA loads increased with increased disease severity^14^. According to our study, the most prevalent strain identified was Boryong then Taguchi. Although the correlation between disease severity and differences of virulence among strains has not been established but more detailed future investigation can lead to better understanding of this.

A previous study reported that for patients aged more than 60 years, absence of eschar, increased WBC count, and decreased albumin are factors indicating severe scrub typhus; the present study demonstrated that the DNA load, in addition to these factors, can also be used as a factor for predicting severe scrub typhus^14^. This study has certain limitations. The samples were collected from only one university hospital, culture test was not perform, and the standardization of quantification methods cannot be ensured since the measured viral load can vary from one laboratory to other.

In the present study, Q-PCR for 16S rRNA exhibited high sensitivity compared to the sensitivities of 56-kDa N-PCR, 47-kDa Q-PCR, and 16S rRNA C-PCR, thus demonstrating that it is a useful test that could be conducted for the early diagnosis of patients suspected to have scrub typhus. Furthermore, the results of Q-PCR can be quantified to obtain copy numbers, which can be used to determine the severity of disease and the risk of complications; therefore, this test will be clinically useful.
